# Comprehensive analysis of the effect of Hashimoto’s thyroiditis on the diagnostic efficacy of preoperative ultrasonography on cervical lymph node lesions in papillary thyroid cancer

**DOI:** 10.3389/fendo.2022.987906

**Published:** 2023-01-12

**Authors:** Hai-Long Tan, AdolphusOsei Nyarko, Sai-li Duan, Ya-Xin Zhao, Pei Chen, Qiao He, Zhe-Jia Zhang, Shi Chang, Peng Huang

**Affiliations:** ^1^ Department of General Surgery, Xiangya Hospital Central South University, Changsha, China; ^2^ Clinical Research Center for Thyroid Disease in Hunan Province, Changsha, China; ^3^ Hunan Provincial Engineering Research Center for Thyroid and Related Diseases Treatment Technology, Changsha, China; ^4^ National Clinical Research Center for Geriatric Disorders, Xiangya Hospital, Changsha, China

**Keywords:** Hashimoto’s thyroiditis, papillary thyroid carcinoma, lymph node metastasis, ultrasonography, diagnostic efficacy

## Abstract

**Purpose:**

Hashimoto’s thyroiditis often leads to reactive hyperplasia of the central compartment lymph nodes in papillary thyroid carcinoma (PTC) patients. However, the effect and clinical significance of Hashimoto’s thyroiditis (HT) on ultrasonography evaluation for cervical lymph node (LN) lesions remain unknown. This study aims to investigate the effect of Hashimoto’s thyroiditis on the diagnostic efficacy of preoperative ultrasonography on cervical lymph node lesions in PTC patients.

**Patients and methods:**

This study consecutively enrolled 1,874 PTC patients who underwent total thyroidectomy and radical cervical lymph node dissection between January 2010 and December 2021. Eligible patients were categorized as with HT and without HT. The diagnostic performance of preoperative ultrasonography for cervical LN lesions (including central LNs and lateral LNs) was evaluated between PTC patients with HT and those without HT, respectively.

**Results:**

Among the 1,874 PTC patients, 790 (42.1%) had central cN+ and 1,610 (85.9%) had lateral cN+. Compared with PTC patients without HT, the preoperative US for central LNs displays a higher false-positive rate (27.9% vs. 12.2%, *p <*0.001) and a lower specificity (72.1% vs. 87.8%, *p* < 0.001) in PTC patients with HT. Moreover, in PTC patients with HT, the ratio of the absence of fatty hilum in central LNs without metastasis was higher than in PTC patients without HT (13.02% vs. 7.46%, *p* = 0.013). However, no such differences were observed in lateral LNs.

**Conclusion:**

HT will interfere with the preoperative US evaluation for central LNs and increase the incidence of the absence of fatty hilum in central benign LNs. When PTC patients have concomitant HT, clinicians should thoroughly evaluate the central LNs, thereby decreasing the incidence of misdiagnosis and unnecessary surgery.

## 1 Introduction

Thyroid carcinoma (TC) is one of the most frequent malignancies with a rapidly rising incidence in the past 40 years worldwide (1, 2). Papillary thyroid carcinoma (PTC) is the most common histologic variant of TC, accounting for approximately 80%–90% (3, 4). Despite PTC being considered a low-grade malignant tumor, lymph node metastasis (LNM) is a common biological behavior and exists in approximately 30%–80% of PTC patients (5–7). Adequate preoperative evaluation and tailoring of surgical resections for primary and metastatic lesions are the most important for PTC patients to minimize the possibility of metastatic lesion residual and recurrence.

In recent years, with the continuous improvement of diagnostic technology, high-resolution ultrasonography (US) has been the most important measure for the preoperative assessment of cervical lymph node metastatic lesions in PTC patients (5, 8, 9). Of note, central lymph node dissection is recommended only for PTC patients with clinically apparent metastatic disease to the nodes (clinical N1) detected by physical examination or preoperative imaging studies or with advanced primary tumors (T3 or T4), according to the 2015 American Thyroid Association (ATA) Management Guidelines (8). However, the diagnostic efficacy of preoperative US on cervical (especially central compartments) lymph node (LN) metastatic lesion is not obvious (10–12).

Hashimoto’s thyroiditis (HT) is the most common autoimmune disease and is frequently accompanied by a humoral immune response and inflammatory manifestations (13–16). Since PTC patients often have concomitant Hashimoto’s thyroiditis (HT), reactive hyperplasia is often observed in the neck LNs, especially central LNs, which may result in some abnormal ultrasonographic features in preoperative imaging examinations (13, 17, 18). However, the effect and clinical significance of HT on ultrasonography evaluation for cervical LN lesions remain unknown.

In the present study, we comprehensively investigate the diagnostic performance of preoperative US on cervical lymph node metastasis lesions and aim to determine the effect of Hashimoto’s thyroiditis on preoperative US for cervical lymph node lesions in papillary thyroid cancer.

## 2 Materials and methods

### 2.1 Patients and study design

**Figure 1:**
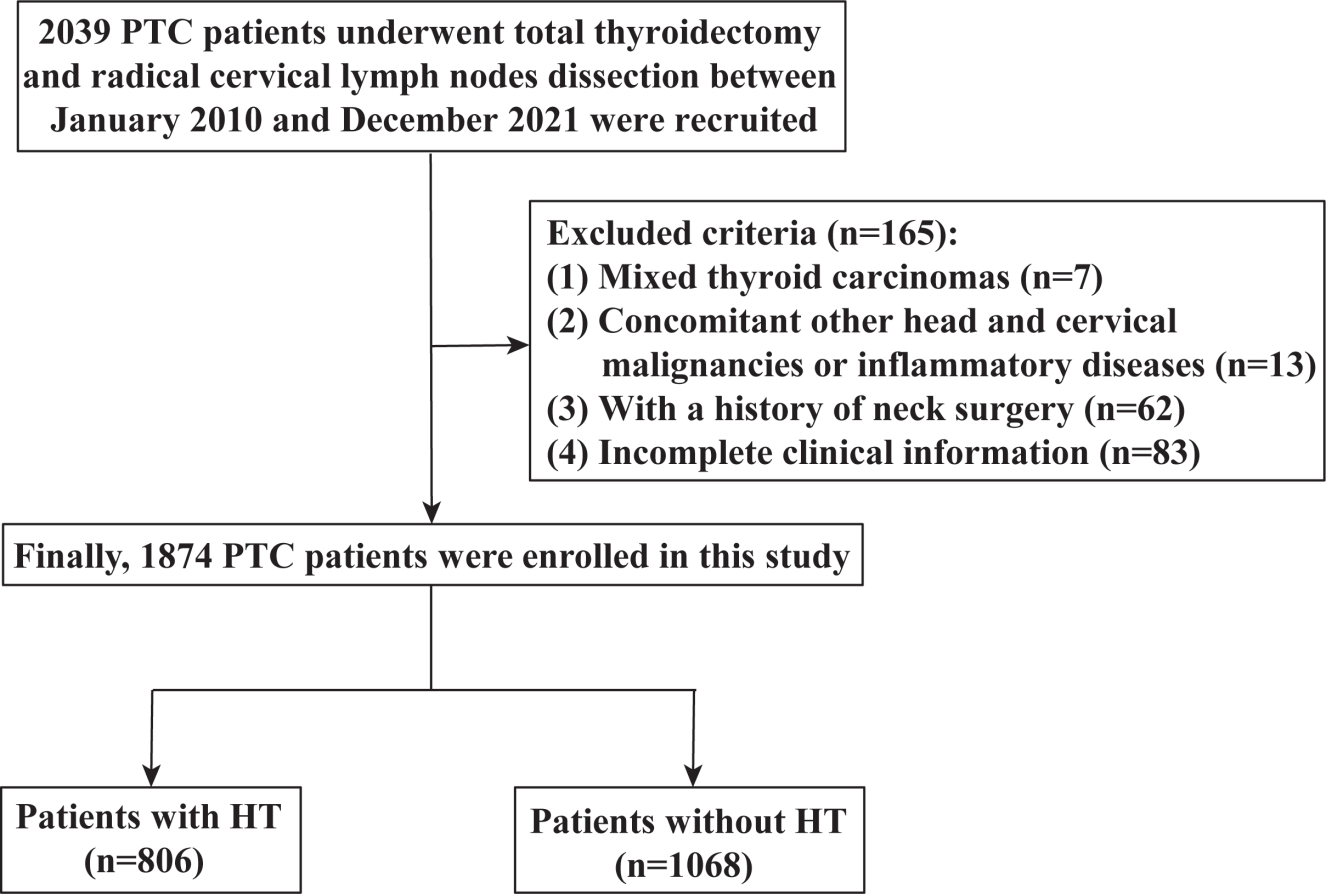
The flowchart of the inclusion and exclusion criteria for the study.

This study was approved by the Ethics Review Committee of Xiangya Hospital, Central South University (20211245) and performed in accordance with the Declaration of Helsinki. The informed consent was waived because of the retrospective and anonymous nature of the study. We enrolled patients who underwent total thyroidectomy and radical cervical lymph node dissection between January 2010 and December 2021 at the Xiangya Hospital, Central South University. All patients underwent physical cervical examination or high-resolution US imaging before surgery. The inclusion criteria were pathologically proven PTC. Patients were excluded if they have any of the following conditions: mixed thyroid carcinomas, PTC combined with other head and cervical malignancies or inflammatory diseases, a history of cervical surgery, and incomplete clinical information. Finally, a total of 1,874 patients with PTC (including 806 patients with HT) were enrolled in this study (**Figure 1**).

### 2.2 Surgical strategy

All patients underwent total thyroidectomy and prophylactic central compartment cervical dissection. Therapeutic lateral lymph node dissection (LLND) was performed exclusively for patients with clinically suspicious lateral LN metastasis, which was confirmed by fine-needle aspiration biopsy and/or US and CT before surgery (8). Prophylactic LLND may be performed for PTC patients with intermediate to high recurrence risk based on the surgeon’s decision and/or the patient’s preferences.

### 2.3 Evaluation of ultrasonography features

All patients received preoperative neck high-resolution ultrasonography examination to evaluate the status of thyroid nodules and cervical central and lateral compartment LNs. Lymph node compartments on ultrasound were separated into levels based on the 2015 ATA management guidelines (7, 8). The abnormal features on ultrasonography suggestive of suspicious LN metastasis included hyperechogenicity, microcalcificationcations, absence of fatty hilum, cystic components, round shape, and peripheral vascularity (8, 19–21). A round shape is defined as a lymph node aspect ratio >0.5. Clinically node-positive (cN+) was defined as with any abnormal ultrasonography features (8), and clinically node-negative (cN−/cNx) was defined as without any abnormal ultrasonography features or LNs undetected on preoperative neck ultrasonography examination (8). The diagnostic accuracy of ultrasonography for LN detection and examination was calculated based on the final pathological findings.

### 2.4 Evaluation of clinicopathological characteristics

The clinicopathological characteristics including age, gender, primary tumor size, extrathyroidal extension, and lymph node metastatic status were collected. Central lymph node metastasis (CLNM) and lateral lymph node metastasis (LLNM) were diagnosed by postoperative histologic examination. Serum antithyroglobulin (TGAb) and antithyroid peroxidase (TPOAb) levels were measured by electrochemiluminescence immunoassay within 7 days before surgery, and the range for normal values was 0–115 IU/ml for TGAb and 0–34 IU/ml for TPOAb. The results were considered positive when TGAb and TPOAb levels exceeded 115 and 34 IU/ml, respectively. HT was diagnosed by postoperative histologic examination or positive preoperative TGAb, TPOA, and diffuse lesions on preoperative ultrasonography (22–25).

### 2.5 Statistical analysis

GraphPad Prism 8.3.1 for Windows (GraphPad Software, San Diego, CA, USA) was used for all statistical analyses. Continuous variables are presented as the mean ± standard deviation (SD), whereas categorical variables are presented as the number of cases with percentages (%). The independent *t*-test was performed for the comparison of continuous variables, and a chi-square test or Fisher’s exact test was conducted for categorical variables. The sensitivity, specificity, positive predictive value (PPV), negative predictive value (NPV), false-positive rate (FPR), and false-negative rate (FNR) for lymph node metastasis diagnosed on ultrasound were calculated based on the patients with and without Hashimoto’s thyroiditis, respectively. The chi-square test was used to compare the results of diagnostic tests (sensitivity, specificity, PPV, NPV, FPR, and FNR) between patients with and without Hashimoto’s thyroiditis. In addition, to further determine the effect of HT in the diagnostic performance of preoperative US for cervical LNs, we analyzed the differences in each ultrasonography feature of LNs between PTC patients with HT and those without HT by the chi-square test. A *p*-value <0.05 was considered statistically significant.

## 3 Results

### 3.1 Compared with patients without HT, abnormal lymph nodes in the central compartment were more easily detected in PTC patients with HT

A total of 1,874 PTC patients were included in the study, consisting of 1,266 women (67.6%) and 608 men (32.4%) (female:male ratio, 2.1:1). In this study, 1,673 patients (89.3%) were younger than 55 years, and 499 (26.6%) patients had a tumor size ≤10 mm. Moreover, 806 (43.0%) patients had concomitant HT, and compared with male PTC patients, HT is prone to occur in female PTC patients (*p* < 0.001).

Among the 1,874 PTC patients, 790 (42.1%) patients had central cN+ and 1,608 (85.9%) patients had lateral cN+, but 709 (89.7%) and 1,363 (84.8%) of them had CLNM and LLNM by histological examination, respectively. In addition, central cN−/cNx and lateral cN−/cNx were found in 1,084 (57.9%) and 266 (14.1%) patients, but 736 (67.9%) and 39 (14.7%) of them had CLNM and LLNM, respectively. Although there was no significant difference in the proportion of CLNM in pathological findings between the patients with HT and without HT (*p* = 0.867), the proportion of CLNM detected on ultrasound (central cN+) was higher in the patients with HT compared with the patients without HT (*p* < 0.001) (**Table 1**).

**Table 1:** Comparison of the clinicopathological characteristics of PTC patients with HT and without HT.

Characteristic	With HT (%)	Without HT (%)	*p*
No. of patients	806 (43.0)	1,068 (57.0)	
Sex			
Male/female	164 (20.3)/642 (79.7)	444 (41.6)/624 (58.4)	**<0.001^a^**
Age (mean ± SD, years)	37.61 ± 11.94	40.13 ± 12.50	**<0.001^b^**
≥55/<55	64 (7.9)/742 (92.1)	137 (12.8)/931 (87.2)	0.520^a^
Tumor size (mean ± SD, mm)	18.28 ± 10.76	18.13 ± 11.40	0.762^b^
≤10/>10	209 (25.9)/597 (74.1)	290 (27.2)/778 (72.8)	0.553^a^
ETE			
Present/absent	260 (32.3)/546 (67.7)	374 (35.0)/694 (65.0)	0.211^a^
Tumor stage			
pT1	401 (49.8)	526 (49.3)	0.172^a^
pT2	132 (16.4)	146 (13.7)	
pT3	171 (21.2)	230 (21.5)	
pT4	102 (12.7)	166 (15.5)	
Clinically node-positive			
Central cN+	386 (47.9)	404 (37.8)	**<0.001^a^**
Lateral cN+	919 (84.5)	929 (87.0)	0.124^a^
CLNM			
Positive/negative	623 (77.3)/183 (22.7)	822 (77.0)/246 (23.0)	0.867^a^
LLNM			
Positive/negative	597 (74.1)/209 (25.9)	805 (75.4)/263 (24.6)	0.519^a^
Number of CLNM (mean ± SD)	3.54 ± 3.61	3.07 ± 3.11	**0.003^c^**
Number of LLNM (mean ± SD)	3.51 ± 3.66	3.26 ± 3.53	0.122^c^
Number of CLND (mean ± SD)	7.95 ± 4.88	5.49 ± 3.88	**<0.001^c^**
Number of LLND (mean ± SD)	16.2 ± 10.11	15.44 ± 9.65	0.095^c^

a The chi-square test was adopted.

b The Student’s t-test was adopted.

c The Mann–Whitney U test was adopted.

Variables with statistical significance are shown in bold.

CLND, central lymph node dissection; CLNM, central lymph node metastasis; ETE, extrathyroidal extension; HT, Hashimoto’s thyroiditis; PTC, papillary thyroid carcinoma; LLND, lateral lymph node dissection; LLNM, lateral lymph node metastasis; SD, standard deviation.

### 3.2 HT decreased the diagnostic efficacy of preoperative US for central metastatic LNs

Furthermore, we compared the diagnostic value of preoperative US for cervical LNs (including central LNs and lateral LNs) between PTC patients with HT and those without HT. Interestingly, compared with those without HT, we found increased sensitivity and FPR of the preoperative US for central LNs in PTC patients with HT (sensitivity, 53.8% vs. 45.5%, *p* = 0.002; FPR, 27.9% vs. 12.2%, *p* < 0.001), but lower specificity, PPV, and FNR (specificity, 72.1% vs. 87.8%, *p* < 0.001; PPV, 86.8% vs. 92.6%, *p* = 0.007; FNR, 46.3% vs. 54.5%, *p* = 0.002) (**Table 2**). Moreover, this difference was also observed when the patients were divided into two groups by tumor size (cutoff = 10 mm) (**Tables S1, S2**). Of note, no differences related to the diagnostic efficacy of the US were observed in lateral LNs (*p* > 0.05) (**Table 3**).

**Table 2:** The diagnostic value for central compartment lymph node metastases in PTC patients with or without HT on neck US.

Parameter	With HT			Without HT		
	CLNM (+) (*n* = 623) (%)	CLNM (−) (*n* = 183) (%)	*p*	CLNM (+) (*n* = 822) (%)	CLNM (−) (*n* = 246) (%)	*p*
Abnormal US	335 (53.8)	51 (27.9)	<0.001^a^	374 (45.5)	30 (12.2)	**<0.001^a^**
Normal US	288 (46.2)	132 (72.1)		448 (54.5)	216 (87.8)	
Value of neck US in the diagnosis of CLNM						
Sensitivity	53.8%			45.5%		**0.002^a^**
Specificity	72.1%			87.8%		**<0.001^a^**
PPV	86.8%			92.6%		**0.007^a^**
NPV	31.4%			32.5%		0.705^a^
FPR	27.9%			12.2%		**<0.001^a^**
FNR	46.2%			54.5%		**0.002^a^**
Accuracy	57.9%			55.2%		0.244^a^

Variables with statistical significance are shown in bold.

HT, Hashimoto’s thyroiditis; CLNM, central lymph node metastasis; FPR, false-positive rate; FNR, false-negative rate; PPV, positive predictive value; NPV, negative predictive value; PTC, papillary thyroid carcinoma; US, ultrasonography.

a The chi-square test was adopted.

**Table 3:** The diagnostic value for lateral compartment lymph node metastases in PTC patients with or without HT on neck US.

Parameter		With HT				Without HT					
		LLNM (+) (*n* = 597) (%)	LLNM (−) (*n* = 209) (%)	*p*		LLNM (+) (*n* = 805) (%)	LLNM (−) (*n* = 263) (%)	*p*			
Abnormal US	580 (97.2)		100 (47.8)	**<0.001^a^**	783 (97.3)		145 (55.1)	**<0.001^a^**		
Normal US	17 (2.8)		109 (52.2)		22 (2.7)		118 (44.9)			
Value of neck US in the diagnosis of LLNM											
Sensitivity	97.2%				97.3%			0.897^a^		
Specificity	52.2%				44.9%			0.116^a^		
PPV	85.3%				84.4%			0.612^a^		
NPV	86.5%				84.3%			0.609^a^		
FPR	47.8%				55.1%			0.116^a^		
FNR	2.8%				2.7%			0.897^a^		
Accuracy	85.5%				84.4%			0.503^a^		

Variables with statistical significance are shown in bold.

HT, Hashimoto’s thyroiditis; LLNM, lateral lymph node metastasis; FPR, false-positive rate; FNR, false-negative rate; PPV, positive predictive value; NPV, negative predictive value; PTC, papillary thyroid carcinoma; US, ultrasonography.

a The chi-square test was adopted.

### 3.3 HT increased the incidence of the absence of fatty hilum in central LNs without metastasis

**Figure 2:**
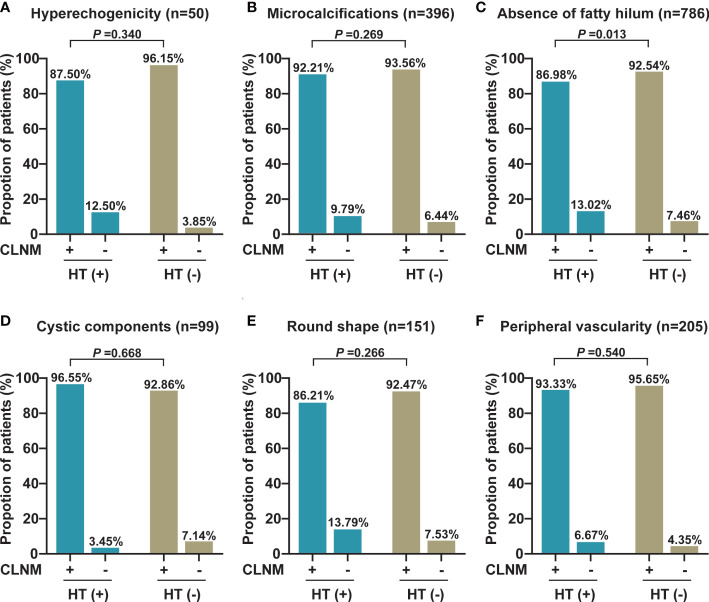
The proportion of each abnormal US feature between malignant and benign central LNs in PTC patients with or without HT. **(A)** The proportion of hyperechogenicity (*n* = 50). **(B)** The proportion of microcalcifications (*n* = 396). **(C)** The proportion of the absence of fatty hilum (*n* = 786). **(D)** The proportion of cystic components (*n* = 99). **(E)** The proportion of round shape (*n* = 151). **(F)** The proportion of peripheral vascularity (*n* = 205). CLNM, central lymph node metastasis; HT, Hashimoto’s thyroiditis.

**Figure 3:**
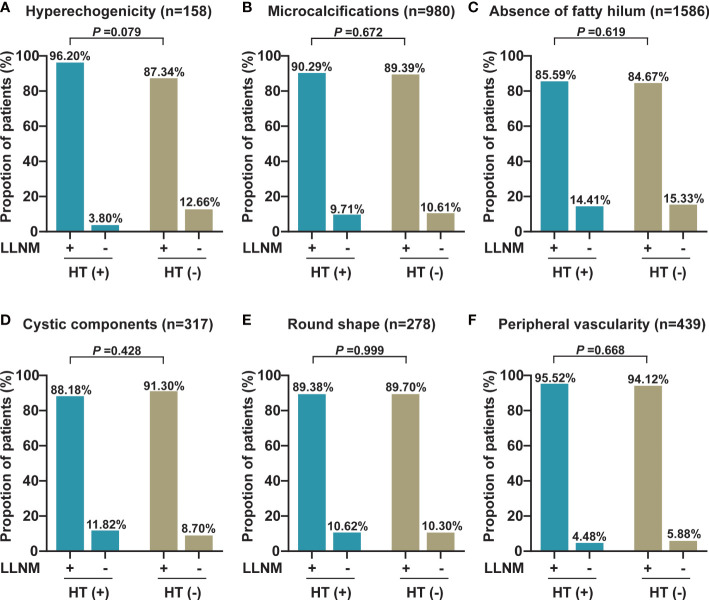
The proportion of each abnormal US feature between malignant and benign lateral LNs in PTC patients with or without HT. **(A)** The proportion of hyperechogenicity (*n* = 158). **(B)** The proportion of microcalcifications (*n* = 980). **(C)** The proportion of the absence of fatty hilum (*n* = 1,586). **(D)** The proportion of cystic components (*n* = 317). **(E)** The proportion of round shape (*n* = 278). **(F)** The proportion of peripheral vascularity (*n* = 439). LLNM, lateral lymph node metastasis; HT, Hashimoto’s thyroiditis.

In addition, we also analyzed the differences in each ultrasonography feature of LNs between PTC patients with HT and those without HT and observed that the absence of fatty hilum of central LNs was more prevalent in the non-metastatic central LNs of PTC patients with HT than in those without HT (13.02% vs. 7.46%, *p* = 0.013). Otherwise, no differences in other ultrasonography features including hyperechogenicity, microcalcifications, cystic components, round shape, and peripheral vascularity were observed (*p* > 0.05) (**Figure 2**). Furthermore, we also compared the lateral lymph node ultrasonography features of PTC patients with HT and without HT, and no significant differences were observed (*p* > 0.05) (**Figure 3**).

## 4 Discussion

This study is the first to comprehensively investigate the effect of HT on the diagnostic efficacy of preoperative US on cervical LNs in PTC patients. Our results indicate that HT will reduce the diagnostic efficacy of preoperative US and increase the misdiagnosis rate of preoperative US for central LNs.

In reality, PTC patients frequently have concomitant HT which manifests as reactive hyperplasia involving the neck LNs especially central LNs, which is frequently observed intraoperatively (17, 18, 26–28). This may interfere with the evaluation of preoperative US for cervical LNs. However, the effect and clinical significance of HT on ultrasonography evaluation for cervical LN lesions remain unknown. Therefore, we comprehensively analyzed the diagnostic efficacy of preoperative US for cervical LNs between PTC patients with HT and those without HT. The results indicate a significantly higher false-positive rate of preoperative US for central LNs in PTC patients with HT as compared with PTC patients without HT (27.9% vs. 12.2%), but this was not observed in lateral LNs. These data suggest an increased misdiagnosis rate of preoperative US for central LNs in PTC patients with HT, which may lead to a surgeon’s misjudgment for central LNs and increase the extent of surgery and the incidence of surgery-related complications.

Preoperative high-resolution US is the primary means for the evaluation of cervical LN lesions by the imaging features of LNs (8). Malignant LNs can be distinguished from normal LNs based on size, shape, echogenicity, hypervascularity, loss of fatty hilum, and calcifications of imaging features (21). Of note, abnormally enlarged LNs are often observed during the preoperative US and intraoperative exploration in PTC patients with HT; however, we often cannot accurately judge the nature of enlarged LNs. Therefore, we compared the differences of each US imaging feature of cervical LNs (including central and lateral compartment LNs) in PTC patients with or without HT. Compared with those PTC patients without HT, we found that the absence of fatty hilum was prone to present on central benign LNs in PTC patients with HT (13.02% vs. 7.46%), but this difference was not observed in lateral compartment LNs. These results denote that clinicians should cautiously evaluate the characteristic of central LNs, when the fatty hilum of central LN was undetected during the US examination in PTC patients with HT, thereby decreasing the possibility of misdiagnosis.

There are some limitations to this study that should be noted. Firstly, although this study found that HT interferes with the evaluation of preoperative US for central LNs in PTC patients, we are currently unable to accurately identify the metastatic status of central LNs in PTC patients with HT before surgery. Secondly, this study lacks external validation, and further studies from multiple centers are needed in the future.

## 5 Conclusion

HT will interfere with the preoperative US evaluation for central LNs and increase the incidence of the absence of fatty hilum in central benign LNs. When PTC patients had concomitant HT, clinicians should thoroughly evaluate the central LNs, thereby decreasing the incidence of misdiagnosis and unnecessary surgery.

## Data availability statement

The original contributions presented in the study are included in the article/**Supplementary Material**. Further inquiries can be directed to the corresponding author.

## Author contributions

Study concepts: H-LT and PH. Study design: H-LT and S-LD. Data acquisition: H-LT and AN. Quality control of data and algorithms: Y-XZ and PC. Data analysis and interpretation: QH and Z-JZ. Statistical analysis: H-LT and Z-JZ. Manuscript preparation: H-LT and PH. Manuscript editing: H-LT, AN, and PH. Manuscript review: H-LT, SC, and PH. All authors contributed to the article and approved the submitted version.
