# Supplementary material for: Comprehensive analysis of the effect of Hashimoto’s thyroiditis on the diagnostic efficacy of preoperative ultrasonography on cervical lymph node lesions in papillary thyroid cancer
Source: Front Endocrinol (Lausanne). 2023 Jan 12;13:987906. doi: 10.3389/fendo.2022.987906 (PMC9877506; doi:10.3389/fendo.2022.987906)
Supplement: Supplementary file 1 [file Table_1.docx]

| Table S1. The diagnostic value for central neck compartment lymph nodes metastases in PTC patients (tumor size ≤10mm) with or without Hashimoto’s thyroiditis on neck US | | | | | | | | | | | | |
| --- | --- | --- | --- | --- | --- | --- | --- | --- | --- | --- | --- | --- |
| **Parameter** | **Hashimoto’s thyroiditis** | | | | | |  | | **Without Hashimoto’s thyroiditis** | | | |
|  | **CLNM (+)**  **(n= 128) (%)** | | | **CLNM (-)**  **(n= 81) (%)** | | ***p*** | | **CLNM (+)**  **(n= 186) (%)** | | **CLNM (-)**  **(n= 104) (%)** | | ***p*** |
| Abnormal US | | 68 (53.1) | | 26 (32.1) | **0.003^a^** | | | 77 (41.4) | | 10 (9.6) | **<0.001^a^** | |
| Normal US | | 60 (46.9) | | 55 (67.9) |  | | | 109 (58.6) | | 94 (90.4) |  | |
| Value of neck US in diagnosis of CLNM | | | | | | | | | | |  | |
| Sensitivity | | | 53.1% | | | | | 41.4% | | | **0.041^a^** | |
| Specificity | | | 67.9% | | | | | 90.4% | | | **<0.001^a^** | |
| PPV | | | 72.3% | | | | | 88.5% | | | **0.006^a^** | |
| NPV | | | 47.8% | | | | | 46.3% | | | 0.794^a^ | |
| FPR | | | 32.1% | | | | | 9.6% | | | **<0.001^a^** | |
| FNR | | | 46.9% | | | | | 58.6% | | | **0.041^a^** | |
| Accuracy | | | 58.9% | | | | | 59.0% | | | 0.980^a^ | |
| **Note:** Variables with statistical significance are shown in bold; the Chi-square test was adopted. | | | | | | | | | | | | |
| **Abbreviations:**  CLNM, central lymph node metastasis; FPR, false positive rate; FNR, false negative rate; PPV, positive predictive value; NPV, negative predictive value; PTC, papillary thyroid carcinoma; US, ultrasonography. | | | | | | | | | | | | |
