# Supplementary material for: Comprehensive analysis of the effect of Hashimoto’s thyroiditis on the diagnostic efficacy of preoperative ultrasonography on cervical lymph node lesions in papillary thyroid cancer
Source: Front Endocrinol (Lausanne). 2023 Jan 12;13:987906. doi: 10.3389/fendo.2022.987906 (PMC9877506; doi:10.3389/fendo.2022.987906)
Supplement: Supplementary file 2 [file Table_2.docx]

| Table S2. The diagnostic value for central neck compartment lymph nodes metastases in PTC patients (tumor size >10mm) with or without Hashimoto’s thyroiditis on neck US | | | | | | | | | | | | |
| --- | --- | --- | --- | --- | --- | --- | --- | --- | --- | --- | --- | --- |
| **Parameter** | **Hashimoto’s thyroiditis** | | | | | |  | | **Without Hashimoto’s thyroiditis** | | | |
|  | **CLNM (+)**  **(n= 495) (%)** | | | **CLNM (-)**  **(n= 102) (%)** | | ***p*** | | **CLNM (+)**  **(n= 636) (%)** | | **CLNM (-)**  **(n= 142) (%)** | | ***p*** |
| Abnormal US | | 267 (53.9) | | 25 (24.5) | **<0.001^a^** | | | 297 (46.7) | | 20 (14.1) | **<0.001^a^** | |
| Normal US | | 228 (46.1) | | 77 (75.5) |  | | | 339 (53.3) | | 122 (85.9) |  | |
| Value of neck US in diagnosis of CLNM | | | | | | | | | | |  | |
| Sensitivity | | | 53.9% | | | | | 46.7% | | | **0.016^a^** | |
| Specificity | | | 75.5% | | | | | 85.9% | | | **0.038^a^** | |
| PPV | | | 91.4% | | | | | 93.7% | | | 0.288^a^ | |
| NPV | | | 22.2% | | | | | 26.5% | | | 0.707^a^ | |
| FPR | | | 24.5% | | | | | 14.1% | | | **0.038^a^** | |
| FNR | | | 46.1% | | | | | 53.3% | | | **0.016^a^** | |
| Accuracy | | | 57.6% | | | | | 53.9% | | | 0.164^a^ | |
| **Note:** Variables with statistical significance are shown in bold; the Chi-square test was adopted. | | | | | | | | | | | | |
| **Abbreviations:**  CLNM, central lymph node metastasis; FPR, false positive rate; FNR, false negative rate; PPV, positive predictive value; NPV, negative predictive value; PTC, papillary thyroid carcinoma; US, ultrasonography. | | | | | | | | | | | | |
